# International and domestic university students’ mental health over the course of the COVID-19 pandemic in Germany: Comparison between 2020, 2021, and 2022

**DOI:** 10.1371/journal.pone.0299812

**Published:** 2024-02-29

**Authors:** Aneliana da Silva Prado, Sabrina Baldofski, Elisabeth Kohls, Christine Rummel-Kluge

**Affiliations:** 1 Department of Psychiatry and Psychotherapy, Medical Faculty, Leipzig University, Leipzig, Sachsen, Germany; 2 Department of Psychology, Federal University of Parana, Curitiba, Parana, Brazil; 3 Campus Curitiba, Federal Institute of Education, Science, and Technology of Parana, Curitiba, Parana, Brazil; 4 Department of Psychiatry and Psychotherapy, University of Leipzig Medical Center, Leipzig, Sachsen, Germany; University of Connecticut Health Center: UConn Health, UNITED STATES

## Abstract

**Background:**

The COVID-19 pandemic affected university students’ mental health worldwide. International students were presenting high levels of stress, anxiety, and depressive symptoms before the pandemic. This study aimed to investigate (i) differences between various timepoints of the COVID-19 pandemic (2020, 2021, and 2022) in mental health outcomes and social and emotional aspects in domestic and international students, separately, (ii) differences between international and domestic students between the three timepoints on mental health outcomes and social and emotional aspects, and (iii) possible moderation effects of timepoints on mental health outcomes and social and emotional aspects of domestic and international students.

**Material and methods:**

Data from three cross-sectional anonymous online surveys conducted in German universities were analyzed and compared. Data were collected in 2020, 2021, and 2022, respectively, with a total *N* = 14,498. Depressive symptoms, hazardous alcohol use, social support, self-efficacy, resilience, perceived stress, and loneliness were assessed through standardized self-report instruments. Differences between domestic and international students in mental health outcomes, and social and emotional aspects across three timepoints were assessed with one-way and two-way ANCOVAs.

**Results:**

Regardless of the timepoint, international students presented more depressive symptoms and perceived stress, lower perceived social support and resilience, but higher levels of self-efficacy and less alcohol consumption compared to domestic students. A significant interaction effect between timepoint and student status emerged only for loneliness.

**Conclusions:**

International students generally presented poorer mental health outcomes than domestic students. Mental health care and prevention such as low-threshold, online counseling should address university students, especially international students.

## Introduction

The COVID-19 pandemic is a health crisis with no precedents, also in terms of its impact on the educational system [1–3]. Besides its negative effects on academic development, the burdens students have been experiencing also affected their mental health [2, 4]. Even before the pandemic, university students presented a higher prevalence of mental health problems in comparison to the general population [5]. Worldwide, studies about university students have shown they were hard hit by the effects of the COVID-19 pandemic [4, 6–12].Pandemic-related increases in depressive symptoms [8, 9, 12], suicidal ideation [8, 9], loneliness and stress [8], alcohol and drug consumption [10], and anxiety [7]are some of the aspects that have been addressed as a point of attention. Nevertheless, while the number of international students seeking a degree outside their home countries has increased over the recent years [13],only a few studies have investigated their mental health specifically [6, 11, 14, 15].

Besides the common-shared burdens that domestic and international university students have faced during the pandemic, international students additionally faced the challenges of adapting to living in a new country, with its new social, cultural, and educational aspects. High levels of acculturative stress and difficulties in adjusting to the host country have been reported even before the COVID-19 pandemic [5, 16]. Therefore, social distancing and isolation experienced by the students due to the host country government’s restriction measures during the pandemic might have had even larger negative effects on their mental health than on their domestic counterparts [11].

Besides the US, Australia, and the United Kingdom, Germany has been one of the most common destinations for international students worldwide and, recently, became the non-English speaking country to receive the highest number of international students from all over the world, being considered the key non-English speaking host country [17]. The number of international students enrolled in German universities achieved the mark of 350,000 students in the winter semester 2021/2022, accounting for 11% of all students enrolled in this semester in Germany [17]. As a comparison, the US, which is the most important host country worldwide, had around 977,000 international students in 2019, but it represented just around 5% of all students enrolled in the country. In Germany, international students represent around 7% of those in bachelor’s programs, 21% of all master’s students, and 25% of doctoral students, and the majority (96%) were seeking a degree at German universities, and not doing temporary study-related mobility [17]. Overall, at least 10% of all university graduates in Germany are from abroad [17]. Because international students account for a relevant proportion of all students in Germany, it is important to investigate their mental health status, especially in comparison to domestic students.

Overall, the COVID-19 pandemic marked a downturn in the number of international students, especially among first-year students, due to travel bans in several countries. In Germany, for most of the time when the borders remained closed, international students were among the exceptions after the first wave of COVID-19 in 2020, being allowed to enter the country to commence their studies in the 2020/2021 semester [17].

Hence, this study aimed to investigate (i) differences between various timepoints of the COVID-19 pandemic (2020, 2021, and 2022) in mental health outcomes and social and emotional aspects in domestic and international students, separately, (ii) differences between international and domestic students between the three timepoints on mental health outcomes and social and emotional aspects, and (iii) possible moderation effects of timepoints on mental health outcomes and social and emotional aspects of domestic and international students. In this study, similarly to Kohls and colleagues [18], we hypothesized that the levels of depressive symptoms, suicidal thoughts, and loneliness would significantly differ between the three points in time, being more prominent in the 2021 cohort due to prolonged restrictions and long-lasting effects of the COVID-19 pandemic for both domestic and international students. We also hypothesized that the levels of depressive symptoms, suicidal thoughts, and loneliness would be higher among international students compared to domestic students.

## Materials and methods

For this study, data from three cross-sectional samples collected by our research group at three different timepoints(i.e., 2020, 2021, and 2022) was used. During each of the three timepoints, an anonymous online survey was conducted, using similar questionnaires across all timepoints. The results of each cross-sectional study have already been published [8, 9, 18].The questionnaires were offered in German and English due to the large number of international students enrolled at German universities who are not proficient in German. From the existing data, in these studies, we performed a secondary analysis by grouping students who answered the online questionnaires in English as “international students”, and who answered in German as “domestic students”. This was because the original studies were not designed to compare these two populations. Nevertheless, considering that international students have been showing poor mental health status and the need to further investigate the impact of COVID-19 on their mental health, as stated previously in the introduction, we used the available data to draw this study. The analysis followed the procedure of a similar study [11].The separate group analyses(one-way ANCOVAS) were conducted to check whether the domestic and international students would present a similar tendency in their results across the three timepoints beyond the differences they would present between them (two-way ANCOVAS).

### Participants and procedures

The first cross-sectional study was conducted in July-August 2020 to investigate the mental health status of university students during the first lockdown in Germany. The sample comprised *N* = 3,382 students enrolled at Leipzig University, Germany [8].

The second cross-sectional study investigating the differences in the mental health status of students between 2020 and 2021 was conducted in March-April 2021, with a total of *N* = 5,642 participating students enrolled at Leipzig University, Germany [9].

The third cross-sectional study was conducted in April-May 2022 to examine the students’ mental health status and identify risk and protective factors relevant to students’ mental health with *N* = 5,474 participants enrolled at six universities in Saxony, Germany, including Leipzig University, Germany [18].

The recruitment procedures were similar in all three studies [8, 9, 18]. Students were contacted via official university email and social media channels of the universities and received an email from the administrative office containing a link to each survey. The inclusion criteria were being enrolled as a university student and being 18 years or older. No exclusion criteria were applied. The Ethics Committee of the Medical Faculty of Leipzig University waived approval for all three studies(2020: on June 22, 2020; 2021: on March 8, 2021; 2022: on November 11, 2021).All participants provided informed consent before participation via an online opt-in function.

As the surveys in 2020 and 2021 were exclusively conducted with students being enrolled at Leipzig University, while the survey in 2022 included six universities in Saxony, Germany, including Leipzig University, Germany, all analyses were repeated including only students from Leipzig University to check for differences in the results. In 2022, students from Leipzig University represented 54.1% (*n* = 2,962) of the sample.

The total sample of this study comprised *N* = 14,498 participants across all three timepoints (2020:*n* = 3,382; 2021:*n* = 5,642; 2022:*n* = 5,474). Of these, *n* = 546 (3.8%) participants completed the questionnaire in English (2020:*n* = 128; 2021:*n* = 173; 2022:*n* = 245) and thus, were considered international students.

### Measures

The measures we analyzed in this study were presented identically in the surveys. Participants could choose to answer in German or English.

### Sociodemographic information

The following sociodemographic information was assessed: gender, age, relationship status, being a parent, residential status, migration background (self or parents),and diagnosis of mental disorder (based on self-report).

### Mental health measures

The Patient Health Questionnaire-9 (PHQ-9) [19] was used to assess depressive symptoms over the last 14 days. It has nine items on a 4-point Likert scale ranging from 0 = “not at all” to 3 = “nearly every day.” The total sum score ranges from 0 to 27, and scores of 10 or more indicate clinically relevant symptoms. Additionally, the presence of suicidal thoughts was assessed through item 9 of the PHQ-9 (“thoughts that you would be better off dead, or of hurting yourself”) with a score of ≥ 1 on a scale from “0 = not at all,” 1 = “several days,” 2 = “more than half the days,” to 3 = “nearly every day.”

Alcohol consumption was assessed using the hazardous alcohol use subscale of the Alcohol Use Disorders Identification Test (AUDIT-C) [20]. It has three items on a 5-point Likert scale ranging from 0 = “never” to 4 = “4 or more times a week” to assess the frequency of participants having alcoholic drinks, the typical quantity they drink when consuming alcohol, and the frequency of heavy alcoholic drinks consumed. The AUDIT-C total sum score ranges from 0 to 12, with higher scores indicating higher alcohol consumption and related risk. Additionally, to assess the frequency of drug consumption, one AUDIT-C item was rephrased to “drug or substance use.”

### Social and emotional aspects

The UCLA 3-Item Loneliness Scale was used to assess experienced loneliness on a 4-point Likert, from 0 = “never” to 3 = “often” [21].The total sum score ranges from 0 to 9 and higher scores indicate more loneliness experienced.

Social support was assessed using the five-item ENRICHED Social Support Inventory (ESSI) [22]. It consists of five items answered on a 5-point Likert scale from 1 = “none of the time” to 5 = “all of the time,” with a total sum score ranging from 5 to 25. Higher scores indicate higher levels of social support.

The 10-item General Self-Efficacy Scale (GSE) was used to explore the general sense of perceived self-efficacy [23]. Items were rated on a 4-point Likert scale ranging from 1 = “not at all true” to 4 = “exactly true;” with a composite sum score ranging from 10 to 40. Higher values indicate higher self-efficacy.

The Brief Resilience Scale (BRS) [24] was used to measure the ability to bounce back or adapt well in the face of adversity, with a 5-point Likert scale ranging from 1 = “strongly disagree” to 5 = “strongly agree.” The scale has three reversely coded items; thus, a scale mean score is computed, with higher values indicating higher levels of resilience.

The perception of stress (PSS-4) was measured using the 4-item Perceived Stress Scale (PSS-4) [25]. Items were answered on a 5-point Likert scale from 0 = “never” to 4 = “very often,” with a total sum score ranging from 0 to 16. Higher scores indicate more perceived stress.

### Statistical analysis

All statistical analyses were performed using IBM SPSS Statistics version 29.0. A two-tailed α = 0.05 was applied to statistical testing.

First, descriptive statistics on sociodemographic information (gender, age, relationship status, residential status, migration background, and diagnosed mental disorder), mental health outcomes(depressive symptoms and hazardous alcohol use), and social and emotional aspects(social support, self-efficacy, resilience, perceived stress, and loneliness)were reported.

Second, to assess differences in continuous outcome variables (age, depressive symptoms, hazardous alcohol use, social support, self-efficacy, resilience, perceived stress, and loneliness), one-way and two-way ANCOVAS were performed. One-way ANCOVAS were used to assess differences between the timepoints (2020, 2021, 2022) on mental health outcomes, and social and emotional aspects within each group of international and domestic students separately. Following, two-way ANCOVAs were used to examine the interaction and possible moderate effects of the timepoint (2020, 2021, 2022) and the student status (international and domestic students)on mental health outcomes, and social and emotional aspects when assessing differences between international and domestic students. Age and being a parent were used as covariates in all analyses as there were significant group differences in these variables depending on the timepoint. Bootstrapping procedure (1000 resamplings; 95%-CI BCa—Bias corrected accelerated) was used to obtain greater reliability of the results, to correct deviations in the normal distribution of the sample, and also to present a 95% confidence interval for the differences between the means [26]. Bonferroni correction for multiple testing was applied. To estimate the effect size for the ANCOVAs, *η*^2^partial was interpreted as small when *η*^2^partial = 0.001, as medium when *η*^2^partial = 0.06, and as large when *η*^2^partial = 0.14 [27].

To assess differences in categorical outcome variables (gender, relationship status, being a parent, residential status, migration background, diagnosed mental disorder, suicidal thoughts, clinically relevant depressive symptoms, and diagnosed mental disorder)*χ*^2^-tests were performed. Where needed, effects found using *χ*^2^-tests were further decomposed by utilizing the *z*-test to compare column proportions. To estimate effect sizes for *χ*^2^-tests, the ϕ coefficient was used, while Cramér’s *V* (ϕ_c_) was used when the contingency table was larger than 2×2, with ϕ, ϕ_c_ = 0.10 indicating a small effect, ϕ, ϕ_c_ = 0.30 an average effect, and ϕ, ϕ_c_ = 0.50 a large effect [27].

As the surveys in 2020 and 2021 were exclusively conducted with students from Leipzig University, while the 2022 survey included six universities in Saxony, Germany, including Leipzig University, Germany, all comparisons were repeated including only students from Leipzig University to check for differences in the results.

## Results

### Sociodemographic information

Sociodemographic information on the total sample for each timepoint is displayed in **Table 1**. Between the timepoints, participants presented significant age differences, being a parent, residential status, income, and student status. Participants were significantly older in the 2020 sample than in 2021 and 2022. More participants who lived alone participated in 2022 than in 2020 and 2021, whereas more participants who did not live alone took part in the 2020 survey. More international students participated in 2022 compared to 2021. There was no difference in gender, relationship status, and migration background between the three timepoints.

**Table 1:** Sociodemographic information of the sample (*N* = 14,498).

Variable, *n* (%)	2020	2021	2022	*F*-test/χ^2^-test	*p*	*η*^2^partial/ϕ_c_
	(*n* = 3,382)	(*n* = 5,642)	(*n* = 5,474)			
Gender				χ^2^(4, 14498) = 5.980	0.201	0.014
Male	967 (28.6)	1642 (29.1)	1,599 (29.2)			
Female	2,374 (70.2)	3,915 (69.4)	3,775 (69.0)			
Diverse	41 (1.2)	85 (1.5)	100 (1.8)			
Age, *M* (*SD*)	23.98 (4.64)^a^	23.47 (4.46)^b^	23.71 (4.81)^c^	*F*(2, 14496) = 13.410	**<0.001**	0.002
Relationship status				χ^2^(2, 14498) = 3.600	0.165	.016
In a relationship	1693 (50.1)^a^	2708 (48.0)^a^	2,668 (48.7)^a^			
Being a parent	189 (5.6)^a^	243 (4.3)^b^	316 (5.8)^a^	χ^2^(2, 14498) = 13.859	**<0.001**	0.31
Residential status				χ^2^(2, 14498) = 28.374	**<0.001**	0.044
Alone	678 (20.0)^a^	1240 (22.0)^b^	1,355 (24.8)^c^			
Migration background				χ^2^(4, 14498) = 7.669	0.104	0.016
Oneself	188 (5.6)^a^^,^^b^	284 (5.0)^b^	338 (6.2)^a^			
Parents	209 (6.2)^a^	364 (6.5)^a^	362 (6.6)^a^			
No migration background	2,985 (88.3)^a^^,^^b^	4,994 (88.5)^b^	4,774 (87.2)^a^			
International student	128 (3.8)^a^^,^^b^	173 (3.1)^b^	245 (4.5)^a^	χ^2^(2, 14498) = 15.233	**<0.001**	0.032
Diagnosed mental disorder	644 (19.0)^a^	1,105 (19.8)^a^	1,239 (22.6)^b^	χ^2^(2, 14444) = 21.065	**<0.001**	0.038

*Note*.Calculation of % from valid cases.

The subscript letters

^a,b,c^ indicate groups that significantly differ from each other.

### Domestic students: Mental health outcomes and social and emotional aspects across 2020, 2021 and 2022

Among the sample of domestic students (*n* = 13,952), one-way ANCOVAs were performed for each dependent variable to test for differences between all timepoints (2020, 2021, and 2022). The results indicated there was a statistically significant effect for timepoint in all measures of domestic students’ mental health outcomes as well as social and emotional aspects (all *p*<0.001). Thus, pairwise comparisons with Bonferroni adjustment for multiple comparisons were performed. The results are presented in **Table 2**.

**Table 2:** Means and confidence intervals of domestic and international students’ mental health outcomes and social and emotional aspects and test results of the differences between the three timepoints (*N* = 14,498).

Variable									
		Domestic students^1^				International students^2^			
		*M* (*SD*)	*95%-CI BCa*	*F*-test/*χ*^2^-test	*η*^2^partial/ϕ_c_	*M* (*SD*)	*95%-CI BCa*	*F*-test/*χ*^2^-test	*η*^2^partial/ϕ_c_
PHQ-9				*F*(2, 13946) = 148.810	*η*^2^partial = 0.021			*F*(2, 541) = 1.586	*η*^2^partial = 0.006
	2020	8.57 (5.39)^a^	8.40–8.74			10.99 (6.41)^a^	9.98–12.00		
	2021	10.05 (8.81)^b^	9.89–10.18			11.43 (6.62)^a^	10.42–12.54		
	2022	8.26 (5.40)^c^	8.10–8.41			10.24 (7.43)^a^	9.31–11.26		
AUDIT-C				*F*(2, 13894) = 9.403	*η*^2^partial = 0.001			*F*(2, 539) = 1.276	*η*^2^partial = 0.005
	2020	2.88 (2.14)^a^	2.81–2.95			2.64 (2.52)^a^	2.25–3.11		
	2021	2.77 (2.19)^b^	2.71–2.83			2.49 (2.17)^a^	2.15–2.82		
	2022	2.94 (2.31)^a^	2.88–3.00			2.29 (2.27)^a^	1.99–2.58		
Drug and substance				*F*(2, 13893) = 21.704	*η*^2^partial = 0.003			*F*(2, 539) = 0.589	*η*^2^partial = 0.002
	2020	1.30 (0.79)^a^	1.27–1.33			1.20 (0.63)^a^	1.11–1.33		
	2021	1.34 (0.86)^a^	1.32–1.37			1.29 (0.75)^a^	1.19–1.41		
	2022	1.24 (0.71)^b^	1.22–1.26			1.25 (0.68)^a^	1.17–1.34		
ESSI				*F*(2, 13946) = 36.048	*η*^2^partial = 0.005			*F*(2, 541) = 1.204	*η*^2^partial = 0.004
	2020	21.52 (3.54)^a^	21.40–21.64			18.38 (5.72)^a^	17.36–19.37		
	2021	20.92 (3.82)^b^	20.83–21.02			17.94 (5.52)^a^	17.16–18.72		
	2022	20.86 (3.96)^b^	20.76–20.97			17.48 (5.41)^a^	16.81–18.15		
GSE				*F*(2, 13951) = 40.666	*η*^2^partial = 0.006			*F*(2, 541) = 1.043	*η*^2^partial = 0.004
	2020	2.86 (0.44)^a^	2.84–2.87			2.90 (0.52)^a^	2.81–3.01		
	2021	2.80 (0.45)^b^	2.79–2.81			2.93 (0.47)^a^	2.86–3.00		
	2022	2.76 (0.46)^c^	2.75–2.78			2.86 (0.52)^a^	2.80–2.92		
BRS				*F*(2, 13891) = 25.755	*η*^2^partial = 0.004			*F*(2, 539) = 2.779	*η*^2^partial = 0.010
	2020	3.16 (0.76)^a^	3.14–3.19			3.08 (0.65)^a^	2.96–3.18		
	2021	3.10 (0.76)^b^	3.08–3.12			3.00 (0.77)^a^	2.90–3.11		
	2022	3.05 (0.78)^c^	3.03–3.06			2.89 (0.73)^a^	2.80–2.99		
PSS-4				*F*(2, 13891) = 66.490	*η*^2^partial = 0.009			*F*(2, 539) = 0.162	*η*^2^partial = 0.001
	2020	7.31 (3.18)^a^	7.21–7.41			8.22 (2.78)^a^	7.75–8.72		
	2021	7.86 (3.09)^b^	7.79–7.94			8.44 (2.83)^a^	8.04–8.86		
	2022	7.18 (3.18)^a^	7.10–7.26			8.36 (3.36)^a^	7.88–8.81		
UCLA-3				*F*(2, 13946) = 1081.362	*η*^2^partial = 0.134			*F*(2, 541 = 46.662	*η*^2^partial = 0.147
	2020	4.55 (1.58)^a^	4.49–4.60			4.39 (1.74)^a^	4.10–4.69		
	2021	6.38 (1.80)^b^	6.34–6.42			5.90 (1.86)^b^	5.63–6.16		
	2022	5.72 (1.85)^c^	5.67–5.77			6.41 (2.05)^c^	6.16–6.67		
Suicidal thoughts		*n*, %		χ^2^(2, 13952) = 41.463	ϕ_c_ = 0.055	*n*, %		χ^2^(2, 546) = .109	ϕ_c_ = 0.014
	2020	456 (14.0)^a^	NA			34 (26.6)^a^	NA		
	2021	888 (16.2)^b^	NA			48 (27.7)^a^	NA		
	2022	1,006 (19.2)^c^	NA			69 (28.2)^a^	NA		
Clinically relevant depressive symptoms				χ^2^(2, 13952) = 235.478	ϕ_c_ = 0.130			χ^2^(2, 546) = 3.918	ϕ_c_ = 0.085
	2020	1,184 (36.4)^a^	NA			65 (50.8)^a^	NA		
	2021	2,651 (48.5)^b^	NA			96 (55.5)^a^	NA		
	2022	1,824 (34.9)^a^	NA			112 (45.7)^a^	NA		
Diagnosed mental disorder				χ^2^(2, 13900) = 20.718	ϕ_c_ = 0.039			χ^2^(2, 544) = 2.088	ϕ_c_ = 0.062
	2020	626 (19.2)^a^	NA			18 (14.1)^a^	NA		
	2021	1071 (19.8)^a^	NA			34 (19.9)^a^	NA		
	2022	1191 (22.8)^b^	NA			48 (19.6)^a^	NA		

*Note*. PHQ-9, Patient Health Questionnaire-9; AUDIT-C, Alcohol Use Disorders Identification Test; ESSI, ENRICHED Social Support Inventory; GSE, General Self-Efficacy Scale; PSS-4, Perceived Stress Scale; UCLA-3, Three-Item Loneliness Scale. Suicidal thoughts based on item 9 ≥ 1 of PHQ-9. BCa–Bias corrected accelerated. NA–Not applicable.

^1^Sample size: 2020, *n* = 3,250; 2021, *n* = 5,417; 2022, *n* = 5,229.

^2^Sample size: 2020, *n* = 128; 2021, *n* = 173; 2022, *n* = 245.

The different superscript letters

^a,b,c^ indicate significant differences between the timepoints(one-way ANCOVAs) within each group of domestic and international students separately based on Bonferroni post-hoc analysis.

When repeating this comparison by analyzing only participants from Leipzig University, where the first two surveys were conducted, there was no difference in the results except for depressive symptoms and drug and substance consumption. For depressive symptoms, while the overall difference remained significant, *F*(2, 11551) = 104.932, *p*<0.001,*η*^2^partial = 0.018, a post-hoc analysis did not find a significant difference in mean scores between the surveys conducted in 2020 and 2022 (*p* = 0.294), while significant differences remained between 2020 and 2021 (*p*<0.001), and between 2021 and2022 (*p*<0.001). For drug and substance consumption, the effect remained significant, *F*(2, 11498) = 10.313, *p*<0.001, *η*^2^partial = 0.002, but the post-hoc analysis showed that a significant difference emerged between2020 and 2021 (*p* = 0.025),yet differences between 2021 and 2022 (*p*<0.001), and between 2020 and 2022 (*p* = 0.032) remained significant.

### International students: Mental health outcomes and social and emotional aspects across 2020, 2021 and 2022

Among the sample of international students (*n* = 546), one-way ANCOVAs were performed for each dependent variable to test for differences between all timepoints (2020, 2021, and 2022).The results indicated there was no statistically significant effect for timepoint in any of international students’ mental health outcomes as well as social and emotional aspects (all *p*>0.05), except in loneliness (UCLA-3; *p*<0.001) (see **Table 2**). Thus, pairwise comparison with Bonferroni adjustment for multiple comparisons was performed only for this variable. Loneliness levels significantly increased from 2020 to 2021 (*p*<0.001), significantly increased from 2021 to 2022 (*p* = 0.011), and significantly differed between 2020 and 2022 (*p*<0.001). When repeating this comparison by analyzing only participants from Leipzig University, the results remained the same.

### Effects of students’ status and timepoint on mental health outcomes and social and emotional aspects

When performing two-way ANCOVAs, the results indicated that the statistically significant effect of student status (domestic and international students)and timepoint (2020, 2021, and 2022) varied according to the variable. The interaction effect (student status* timepoint) was statistically significant only for loneliness (UCLA-3). Detailed results are presented as follows.

First, there was a statistically significant effect of student status (domestic and international students) on the following dependent variables: depressive symptoms (PHQ-9), *F*(1, 14489) = 64.216, *p*<0.001, *η*^2^partial = 0.004; alcohol consumption (AUDIT-C), *F*(1, 14435) = 16.840, *p*<0.001, *η*^2^partial = 0.001; social support (ESSI), *F*(1, 14489) = 300.373, *p*<0.001, *η*^2^partial = 0.020; self-efficacy (GSE), *F*(1, 14489) = 17.546 *p*<0.001, *η*^2^partial = 0.001; resilience (BRS), *F*(1, 14432) = 8.982, *p* = 0.003, *η*^2^partial = 0.001; perceived stress (PSS-4), *F*(1, 14432) = 39.597, *p*<0.001, *η*^2^partial = 0.003. These results indicate that regardless of the timepoint, international students presented higher levels of depressive symptoms, lower perceived social support, lower levels of resilience, and higher levels of perceived stress compared to domestic students. On the other hand, domestic students consumed alcohol more frequently and presented lower levels of self-efficacy compared to international students regardless of the timepoint. There was no difference between the two groups in perceived loneliness (UCLA-3; *F*(2, 14489) = 0.759, *p* = 0.384, *η*^2^partial = 0.000), neither in drug and substance consumption, *F*(2, 14434) = 2.676, *p* = 0.102, *η*^2^partial = 0.000).When repeating this comparison by analyzing only participants from Leipzig University, results remained the same for all variables, except resilience (BRS), which did not show significant effects(*F*(2,11920) = 4.544, *p* = 0.033, *η*^2^partial = 0.000).

Second, there was a statistically significant effect of the timepoint (2020, 2021, and 2022) for the following dependent variables: depressive symptoms (PHQ-9), social support (ESSI), resilience (BRS), and loneliness (UCLA-3). Thus, pairwise comparisons with Bonferroni adjustment for multiple comparisons were performed for those variables as follows.

For depressive symptoms (PHQ-9), *F*(2, 14489) = 13.559, *p*<0.001, *η*^2^partial = 0.002, levels significantly increased from 2020 to 2021 (*p* = 0.017), while significantly decreased from 2021to 2022 (*p*<0.001), but did not differ between 2020 and 2022 (*p* = 0.224).

For social support (ESSI), *F*(2, 14489) = 6.746, *p*<0.001, *η*^2^partial = 0.001, levels did not differ from 2020 to 2021 (*p* = 0.056), neither from 2021 to 2022 (*p* = 0.602), while significantly decreased from 2020 to 2022 (*p*<0.001).

For resilience (BRS), *F*(2, 14432) = 6.753, *p*<0.001, *η*^2^partial = 0.001, levels did not differ from 2020 to 2021 (*p* = 0.391), neither from 2021 to 2022 (*p* = 0.096), while significantly decreased from 2020 to 2022 (*p*<0.001).

For loneliness (UCLA-3), *F*(2, 14489) = 156.706, *p*<0.000, *η*^2^partial = 0.021, levels significantly increased from 2020 to 2021 (*p*<0.001), did not differ from 2021 to 2022 (*p* = 1.000), while significantly increased from 2020 to 2022 (*p*<0.001).

No difference was found between the timepoints for alcohol consumption (AUDIT-C; *F*(2, 14435) = 0.786, *p* = .456, *η*^2^partial = 0.000), self-efficacy (GSE; *F*(2, 14489) = 4.503, *p* = 0.011, *η*^2^partial = 0.001), perceived stress (PSS-4;*F*(2, 14432) = 3.323, *p* = 0.036, *η*^2^partial = .000), and drug and substance consumption (*F*(1, 14434) = 1.809, *p* = 0.164, *η*^2^partial = 0.000). When repeating this comparison by analyzing only participants from Leipzig University, the results remained the same for all the measures.

Third, there were no statistically significant effects for the interaction timepoint*student status in all the measures (all *p*>0.05), except for loneliness (UCLA-3), *F*(2, 14489 = 23.617, *p*<0.001, *η*^2^partial = 0.003). Therefore, pairwise comparisons to evaluate the interaction effects (timepoint*students status) were performed with Bonferroni adjustment for multiple comparisons only for this variable. In 2020, there was no difference between domestic and international students (*p* = 0.522). In 2021, domestic students presented significantly higher perceived loneliness than international students (*p* = 0.002), whereas in 2022 international students scored higher for loneliness than domestic students (*p*<0.001).

Among domestic students, the pairwise comparison showed there were significant differences in UCLA-3 mean scores between all the timepoints (all *p*<0.001). In 2021, domestic students presented the highest mean score for loneliness. Among international students, the increase in loneliness also significantly differed between 2020 compared to 2021 and 2022 (both *p*<0.001), and between 2021 and 2022 (*p* = 0.009). These results are similar to the one-way ANCOVAs reported earlier. When repeating this comparison by analyzing only participants from Leipzig University, the results remained the same for all measures.

Furthermore, results of chi-square analyses for the categorial variables indicated that international students were more likely to present suicidal thoughts than domestic students in 2020,χ^2^(1, 3382) = 15.654, *p*<0.001, ϕ = 0.068; 2021, χ^2^(1, 5642) = 16.051,*p*<0.001,ϕ = 0.053; and 2022 χ^2^(1, 5474) = 11.811, *p*<0.001, ϕ = 0.046. They also presented clinically relevant depressive symptoms more frequently than domestic students in 2020, χ^2^(1, 3382) = 10.957, *p*<0.001, ϕ = 0.057, and in 2022, χ^2^(1, 5474) = 12.013, *p*<0.001, ϕ = .047, but not in 2021 (*p* = 0.069).No significant difference was found in the number of domestic and international students reporting a diagnosed mental disorder in 2020 (*p* = 0.144), 2021 (*p* = 0.971), and 2022 (*p* = 0.244).When repeating these comparisons by analyzing only participants from Leipzig University, the results remained the same for all these variables.

## Discussion

This study aimed to investigate differences between international and domestic students in mental health outcomes and social and emotional aspects across three timepoints during the COVID-19 pandemic in Germany (2020, 2021, 2022).As hypothesized, the levels of depressive symptoms, suicidal thoughts, and loneliness significantly differed across the three points in time, but only among domestic students. Among international students, mental health outcomes did not differ across time, except for loneliness. Specifically, domestic students presented the highest levels of depressive symptoms and loneliness in 2021, but suicidal thoughts were most prevalent in 2022. As expected, levels of depressive symptoms and suicidal thoughts were higher among international students compared to domestic students regardless of the timepoint. International students presented higher levels of loneliness in 2021 and 2022 compared to 2020. Surprisingly, in 2021, they presented lower perceived loneliness than domestic students.

The results indicate that negative effects of the COVID-19 pandemic were present in both groups of students, but in different ways: while domestic students had their mental health and social and emotional aspects worsened during the COVID-19 pandemic, international students presented a poorer mental health status overall. It indicates that mental health care should address both groups, with special attention on international students, taking into consideration the specificities they may present [28].

Regardless of the timepoint, international students presented more depressive symptoms and perceived stress, lower perceived social support and resilience, but higher levels of self-efficacy compared to domestic students. These results corroborate previous findings indicating that while domestic students had their mental health worsened by the COVID-19 pandemic, international students were less affected as they had already poorer mental health outcomes from the beginning [11, 29]. Among international students, mean scores of depressive symptoms reflected clinically relevant depressive symptoms for all timepoints, whereas domestic students presented a mean score indicating clinically relevant depressive symptoms only in 2021. Our results can be compared to a longitudinal study in China that found significantly more depressive symptoms among international students in the so-called post-pandemic (October to December 2021) assessment compared to the pre-pandemic (December 2019 to April 2020) timepoint [30].

While the severity of depressive symptoms decreased among domestic students in 2022 compared to 2021 and 2020, a higher prevalence of suicidal thoughts was observed in 2022. Further research is needed to understand this result better. However, as discussed by Kohls and colleagues [18], we believe that the number of deaths by COVID-19, and the socioeconomic and political context of the Russian-Ukrainian war, may have affected the prevalence of suicidal thoughts among domestic students. Also, as a complex and multifactorial phenomenon, it could be understood as an indication of an accumulative increase in overall risk factors for suicide related to the outcomes of the most challenging health crisis of our times, the COVID-19 pandemic (e.g., increased stress, uncertainty, fear, and feelings of hopelessness and helplessness) [18, 31]. As the disclosure of suicidal ideation and behaviors (thoughts, plans, or attempts) may prevent dying by suicide [32], this is an important finding to be taken into consideration in mental health promotion actions targeting domestic students.

While a non-significant difference was found between the timepoints among international students, they were more likely to present suicidal thoughts than domestic students in 2020, 2021, and 2022. Our findings are in line with previous studies indicating higher depressive symptoms [11, 29] and suicidal thoughts [11] among international students in comparison to their domestic counterparts. The prevalence of clinically relevant depressive symptoms observed in our sample among international students across the timepoints (2020: 50.8%, 2021: 55.5%, and 2022: 45.7%) was lower than in China, where 61.9% of international students presented moderate-to-severe depressive symptoms in 2020 [33]. Nevertheless, it was higher than in the US, where 24.5% of international students presented moderate-to-severe depressive symptoms in 2020 [34], and in South Korea, where 15% of international students presented clinically relevant depressive symptoms in 2020 [35].

Domestic students consumed alcohol more frequently compared to international students at all timepoints. This result differed from a previous study conducted in the Netherlands [11] which found no difference in alcohol consumption between domestic and international students. Data on alcohol consumption among university students is heterogeneous [18], and further research is necessary to understand the differences between domestic and international students concerning alcohol consumption.

Despite the decrease in the levels of social support observed among domestic students from 2020 to 2021 and 2022, they presented higher levels of social support compared to international students in all three timepoints evaluated. This was not surprising when considering that international students leave their families and social support systems behind when moving to the host country [11]. Lower social support among international students in our sample is consistent with previous findings [29, 36–38]. Moreover, language barriers, lack of knowledge of the host country’s health care system, and stigma associated with mental health services may hinder help-seeking [39].

While the levels of self-efficacy in domestic students decreasedin2021 and 2022compared to the previous timepoint, no significant difference was observed in self-efficacy among international students between the timepoints. Interestingly, international students presented higher levels of self-efficacy than their domestic counterparts in 2020, 2021, and 2022. It might be related to the need for international students to manage their studies and take care of themselves while not having as much social support available as domestic students. A recent report showed that international students usually already have higher education experience in their country of origin when they come to Germany (e.g., 50% have already obtained a first degree abroad). Also, a study before the pandemic indicated that international students had high self-efficacy in most academic tasks [40].These are interesting findings because self-efficacy is a relevant aspect of academic performance.

Domestic students had their levels of resilience decreasing in 2021 and 2022 compared to the respective previous timepoints. Differently, no differences in resilience levels across the timepoints were found among international students. In 2020, 2021, and 2022 domestic students presented higher resilience levels than international students. Because resilience isa protective factor for the severity of anxiety and depression among university students [18, 36], and also for academic burnout among international students in the so-called authors’ “post-COVID-19 new normal (Fall semester 2022–2023)” [41], these results could be related to the higher levels of depressive symptoms among international students.

While domestic students presented higher sum scores of perceived stress in 2021 compared to 2020 and 2022, no significant changes were observed in levels of perceived stress among international students across the timepoints. Nevertheless, regardless of the timepoint, international students presented higher perceived stress compared to domestic students, indicating their levels of perceived stress were already higher from the first timepoint assessment. These results are in line with previous studies indicating a high prevalence (more than 80%) of moderate-to-high perceived stress among international students in the UK and US [36].

Despite the common-shared burdens of the COVID-19 pandemic that domestic and international students faced, international students presented overall poorer mental health outcomes compared to domestic students. For example, in Australia, international students reported race-based discrimination during the COVID-19 pandemic [29]. Concerns about visa status and consequent disruption of studies were reported by international students in the US [42]. A qualitative study conducted in China indicated hopelessness, uncertainty, worry, lack of interest and focus, lack of support, financial difficulties, social pressure, sleep disorders, and increased smoking among international students [28]. Therefore, visa and financial issues, language skills, acculturation problems, poor social support systems, cultural and social adjustment, academic adaptation, homesickness, and lack of knowledge about the healthcare system are some examples of the challenges faced by international students in particular [17, 43–46].

Considering the increase in international students’ mobility—e.g., 6.1 million students were enrolled outside their home country in 2019 [17]—proactive initiatives could be taken to prevent and promote their mental health, even beyond the COVID-19 pandemic[43]. In Germany, a sense of belonging has been pointed out as beneficial for international students’ adaptation and has been related to better well-being, higher study satisfaction, and lower drop-out intention [44]. Furthermore, poor linguistic proficiency, financial problems, a lack of social and academic integration, and misconceptions regarding the teaching and learning culture at German higher education institutions have been indicated as some of the main reasons for international students to dropout of German universities [47].

In China, offline activities, physical exercising, and classroom activities helped to relieve anxiety among international students [48]. Besides, counseling, student support groups, and psychosocial and academic support are suggested to help students to cope with their burdens [36, 42]. Considering that drop-out rates are higher among international students than among domestic students [17], such actions are beneficial in terms of mental health, may prevent drop-out of studies, and even enhance students’ academic performance. Additionally, low-threshold programs such as online interventions (e.g., counseling, support groups, applications) tailored according to international students’ needs are suggested to be developed as it could facilitate help-seeking behavior by diminishing barriers such as stigma and insufficient knowledge of the health care system functioning, develop coping strategies, and increase the sense of university belonging.

## Strengths and limitations

This study presents some strengths. Firstly, by examining the three timepoints during the COVID-19 pandemic in several German Universities with large sample sizes, these results may contribute to the literature on the long-lasting effects of the pandemic on both domestic and international university students’ mental health. Second, to our knowledge, this is the first study to compare domestic and international students in Germany through a two-year evaluation with three timepoints (2020 to 2022). Finally, with the increase in international mobility, our results may contribute to the action call to policymakers and universities to provide proactive mental health promotion programs to their international students in Germany and worldwide. This study presents also some limitations. Firstly, the differences between sample sizes for domestic and international students are considered to be representative of their respective university student populations in Germany. To minimize the sample size differences, we used a bootstrapping procedure and presented the 95%-CI BCa [26]. Second, because we performed a secondary analysis from existing data, we did not assess the participants’ origin and grouped students who answered the online questionnaires in English as international students—therefore, we could not investigate whether there were differences in students’ mental health status depending on their nationalities/cultures and differences/proximity to the host country. Although there might be some students who were international and answered in German, and domestic students who might have answered in English (though unlikely),the results were consistent throughout the analysis and comparable to existing literature, so they can be considered a good approximation. We suggest further research to include more details about the culture and nationality of the students (e.g., questions about the participant’s country of origin/nationality), which could help in identifying subgroups of more vulnerable students who might need more counseling and mental health support. Third, although the study includes three cross-sectional studies, two of them were based entirely on the data from Leipzig University. Accordingly, the results could vary if multiple universities/sites were involved in all three cross-sectional studies, which could be investigated in further research being conducted in several German regions. Finally, this was not a longitudinal study and because of the anonymity of the surveys, we could not indicate how many participants answered the surveys one or more times, so no causal relation can be addressed based on the results. Longitudinal studies are indicated to foster knowledge about international students’ mental health. Nevertheless, these results may shed some light on building mental health promotion and care measures targeting international students.
